# Effect of moderation on rubric criteria for inter-rater reliability in an objective structured clinical examination with real patients

**DOI:** 10.20407/fmj.2021-010

**Published:** 2021-11-25

**Authors:** Tetsuro Watari, Soichiro Koyama, Yusaku Kato, Yonho Paku, Yoshikiyo Kanada, Hiroaki Sakurai

**Affiliations:** 1 Faculty of Rehabilitation, School of Health Sciences, Fujita Health University, Toyoake, Aichi, Japan; 2 Department of Rehabilitation, Tomita Hospital, Okazaki, Aichi, Japan

**Keywords:** OSCE, Video assessment, Moderation, Inter-rater agreement, Real patients

## Abstract

Objectives: Objective structured clinical examinations (OSCEs) are used to assess clinical competence in medical education. Evaluations using video-recorded OSCEs are effective in reducing costs in terms of time and human resources. To improve inter-rater reliability, these evaluations undergo moderation in the form of a discussion between the raters to obtain consistency in grading according to the rubric criteria. We examined the effect of moderation related to the rubric criteria on the inter-rater reliability of a video-recorded OSCE with real patients.

**Methods:**

Forty OSCE videos in which students performed range-of-motion tests at shoulder abduction on real patients were assessed by two raters. The two raters scored videos 1 to 10 without moderation and videos 11 to 40 with moderation each time. The inter-rater reliability of the OSCE was calculated using the weighted kappa coefficient.

**Results:**

The mean scores of the weighted kappa coefficients were 0.49 for videos 1 to 10, 0.57 for videos 11 to 20, 0.66 for videos 21 to 30, and 0.82 for videos 31 to 40.

**Conclusions:**

An assessment of video-recorded OSCEs was conducted with real patients in a real clinical setting. Repeated moderation improved the inter-rater reliability. This study suggests the effectiveness of moderation in OSCEs with real patients.

## Introduction

The objective structured clinical examination (OSCE), first proposed by Harden,^1^ is an objective method for assessing clinical competence (e.g., medical skills and attitudes toward patients) of healthcare professionals in clinical settings. The OSCE has been used to evaluate medical students since the 1970s^2^ and is widely adopted by health professionals such as nurses, pharmacists, radiologists, physical therapists, and occupational therapists.^3–7^

The OSCE evaluates the student’s actual performance. Previous studies have also focused on OSCEs that evaluate student performance recorded using video. Vivekananda-Schmidt et al.^8^ suggested that video OSCEs offer considerable potential advantages to examiners. An OSCE for a group of students is very time-consuming and requires a high level of clinical expertise and coordination. Videotaping the student’s performance and marking the performance at a later time point allows the OSCE to be run with relatively few examiners because stations do not necessarily have to be manned by examiners.^8^ The cost and stress involved in organizing the OSCE is reduced while improving the consistency and fairness of assessments.^8^

Although the OSCE has mainly been conducted on students at educational institutions, some studies have assessed the efficacy of conducting the OSCE with real patients in clinical settings instead of simulated patients.^9,10^ Simulated patients need to exhibit the behavior and communication of real patients because they are required to behave like real patients during an OSCE.^11^ Real patients can provide an adequate opportunity to assess a candidate’s skills. Patients’ views of their participation in high-risk clinical examinations have been reported as favorable.^9,10^ However, one study showed that standardization is difficult because patients’ symptoms tend to change with each examination; moreover, their physical functions are not uniform even if they have the same disease.^12^ The effect of moderation among raters on the reliability of the OSCE may be due to inter-rater sharing of patient information.

Some studies have used various methods of rater training^13–15^ to improve the inter-rater reliability of the OSCE. Pell et al.^13^ reported that the inter-rater reliability of the OSCE was improved by training raters on the scoring criteria. Holmboe et al.^14^ reported that training in OSCE assessment (e.g., mini-lectures, interactive small groups, videotape assessment, and practice of assessment skills with standardized residents and patients) improved the consistency of the OSCE scores. Likewise, Lin et al.^15^ reported that training for OSCE assessment (e.g., discussions and role-play among raters) improved the inter-rater reliability of the OSCE.

Rubric assessments have been used to standardize the scoring of raters in the OSCE.^16^ A rubric is a document that articulates the expectations for an assignment, or a set of assignments, by listing the assessment criteria and describing levels of quality in relation to each of these criteria.^17^ Rubrics can be used to validly assess performance because they are accompanied by detailed scoring guides.^18,19^

Moderation has also been used to improve the reliability of rubric assessments. Moderation is the formal process by which raters discuss the acquisition of consistency and agreement when grading using rubric assessments.^20^ Scoring agreement among the raters is important for improving the inter-rater reliability of the OSCE. Moni et al.^21^ reported that moderation of the rationale for the assessment improved inter-rater agreement.

In the education of physiotherapists and occupational therapists, conducting OSCEs with real patients is necessary. Educational methods for supervisors should be developed to improve inter-rater reliability of OSCEs in the clinical setting. Previous studies have shown that using a rubric to perform moderation among raters is crucial for improving the inter-rater reliability of the OSCE.^20,21^

However, no reports have described the impact of moderation on the inter-rater reliability of the OSCE in students assessed by physical and occupational therapists for real patients in a clinical setting. The primary purpose of this study was to examine the effect of moderation associated with each rubric criterion on the inter-rater reliability of OSCEs for real patients in clinical settings.

## Methods

### Participants

This study was conducted by one certified physical therapist and one certified occupational therapist with at least 9 years of clinical experience in a hospital setting. The study was based on protocols from previous studies^15,22–28^ examining inter-rater reliability and validity of OSCEs. Therapists with 9 years of experience have the highest frequency in Japan and comprise the largest occupational population. In addition, most therapists in Japan have only a few years of experience.^29^ This indicates the need to train the next generation as an organizational unit. Therefore, therapists with more than 9 years of experience were selected for this study because they are expected to play this role.

The study protocol was approved by the ethics review committee of our university (HM17-144). Both therapists provided written informed consent before participating in the study. All patients gave written informed consent. The study was performed in accordance with the Declaration of Helsinki.

### OSCE

The OSCE was conducted based on a textbook for students of rehabilitation therapy written in Japanese.^30^ The scoring rubric consists of 3 attitude items and 14 skill items with scores ranging from 0 to 2. The OSCE task was to measure the shoulder abduction range of motion of the upper limb on the paralyzed side for patients with hemiplegic stroke. The procedure for this task was as follows: greet the patient, who was waiting in a sitting position; explain the test methodology; evaluate the range of motion of the joints (active and passive); test for pain; measure the joint angles; and report the results. The details are presented in Table 1. The maximum score was 28 points and the minimum score was 0 points.

### OSCE videos

Forty OSCE videos were recorded for evaluation. Each OSCE video showed the measurement of the range of motion of the shoulder joint in real patients with hemiplegic stroke by students of physical and occupational therapy during their clinical internship. All patients had limited range of motion in shoulder abduction on the paralyzed side, and all were able to communicate and sit independently. The OSCE video was recorded using two Apple iPads (Apple Inc., Cupertino, CA, USA). One iPad was used to capture the entire patient from the front, while the other was used to capture the student’s entire performance as the photographer moved around.

### Experimental procedure

The 40 randomly selected OSCE videos were scored by 2 raters using a scoring rubric. The two raters scored the OSCE videos using a computer screen located between the two raters. Each video was viewed twice. The first 10 videos were viewed consecutively: the first time for 5 minutes of viewing and 2 minutes of scoring, and the second time for 5 minutes of viewing and 1 minute of scoring. The latter 30 videos consisted of a series of 5 minutes for the first viewing, 2 minutes of scoring, 5 minutes for the second viewing, 1 minute of scoring, and 5 minutes of discussion for moderation.

The moderation was performed using the following steps. (1) The raters verbally reconfirmed how the students performed in the video. (2) The raters explained to each other why each grader gave the video a score of 0 or 1. (3) The two raters made a final decision on what score to give the video.

### Data analysis

The scoring agreement between the two raters was determined using the weighted kappa coefficient. The average kappa coefficients were calculated for videos 1 to 10, which were not discussed between the raters. Videos 11 to 40 were divided into 10 video categories (11–20, 21–30, and 31–40), and the average kappa coefficients were calculated. After one-way analysis of variance, each mean value was compared using Dunnett’s test for multiple comparisons. IBM SPSS, Version 26 (IBM Corp., Armonk, NY, USA) was used for the analysis, and the significance level was set at 5%.

## Results

**Figure 1:**
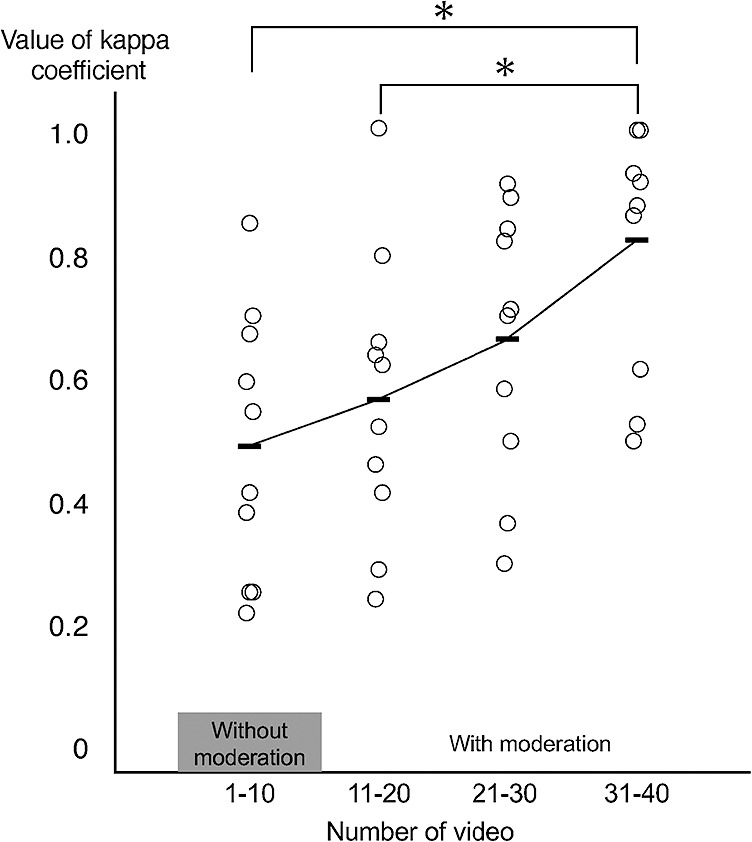
Kappa coefficients between the two raters for all 40 videos. The line plot shows the average value for each set of 10 videos; the first 10 videos were evaluated without moderation.

The scoring agreements for videos 1 to 40 are shown in Table 2. The mean score agreement coefficient for the videos without discussion (videos 1–10) was 0.49. The mean score agreement coefficient for the videos with discussion was 0.57 for videos 11 to 20, 0.66 for videos 21 to 30, and 0.82 for videos 31 to 40. The multiple comparison tests showed that the mean coefficients of agreement between the scores for videos 1 to 10 and videos 11 to 20 were predominantly lower than that of videos 31 to 40 (Figure 1).

## Discussion

This study examined the effect of moderation on the inter-rater agreement of the OSCE. The raters scored the students’ OSCE videos involving real patients and then moderated each OSCE video. Moderation is a process that involves a discussion between raters that enables the raters to arrive at a common understanding of the scoring criteria. Gipps^13^ described the necessity of moderation in the theory of educational evaluation. The results of this study suggest that moderation improves scoring agreement among raters. Liao et al.^31^ reported that although inter-rater agreement and inter-rater reliability are not always the same, improved inter-rater agreement allows students to receive stable scores from different raters.

In the present study, the kappa coefficient increased from 0.49 to 0.82 because of repeated moderation between the raters. Landis and Koch^32^ defined a kappa value of 0.41 to 0.6 as moderate and 0.81 to 1.0 as very good. Lyngå et al.^33^ reported that the inter-rater reliability of the OSCE for nursing students and faculty was high, with a kappa coefficient of 0.79. Borders et al.^34^ reported that the inter-rater reliability of the laryngeal sensation test improved with consensus training of the raters from a kappa factor of 0.22 to 0.42. Moderation is a discussion that is conducted to reach a consensus on the rating scores given by the raters. Through repeated agreement on the scores, the scoring criteria are shared among the raters, and the kappa coefficient, which indicates the degree of agreement on the scoring, is expected to gradually improve.^35,36^

This study examined the effect of moderation on inter-rater agreement. The inter-rater agreement for the unmoderated video ratings was 0.49. The inter-rater agreement of 20 or more video ratings for videos 31 to 40 was 0.82. These results suggest that repeated moderation is necessary to improve inter-rater agreement. Gawad et al.^37^ reported an intraclass correlation coefficient (ICC) of 0.26 for the inter-rater reliability of five raters evaluating three surgical videos, compared with an ICC of 0.76 when two raters evaluated 33 surgical videos. Lou et al.^38^ reported that after 30 minutes of moderation, the inter-rater agreement of student surgical videos improved from an ICC of 0.76 to 0.9. These reports suggest that time-consuming and repeated moderation is required to improve agreement between raters.

In this study, we used student OSCE videos as an evaluation tool. Evaluation using videos was possible and could be used as training through moderation in this study. Washino et al.^39^ suggested the use of video recordings of actual OSCEs as an evaluation training method for OSCEs. Ohyama et al.^40^ reported that scoring using videos taken of OSCEs showed 89.5% agreement among raters. This study also suggested that OSCEs conducted by students on actual patients could be implemented. Collins and Harden^12^ stated that the use of real patients is easy, requires no additional resources or organizational support, and can be a reliable clinical experience.

This study had some limitations that need to be mentioned. First, the OSCE was performed only for one range-of-motion measurement task. Therefore, it is necessary to confirm the results by performing OSCEs for different tasks. Second, the sample size was small; there were only two raters. It is necessary to verify whether the same result can be obtained by increasing the number of raters. Third, this study was conducted in a clinical setting; however, the OSCE is usually performed in schools and uses simulated patients. Therefore, it is necessary to conduct research to verify the effects of the OSCE in educational institutions. Fourth, the order in which the 40 OSCE videos were used was not considered. Sturpe et al.^41^ performed a study of OSCEs in video evaluations and found that raters’ memory bias was unlikely to have an effect on the outcome of the evaluation. Further research should be performed to determine whether similar results are obtained when the order in which the videos were used in this study is changed.

In this study, students performing OSCEs with real patients in a real clinical setting were video-evaluated by clinically engaged physical and occupational therapists. In clinical OSCEs, it is possible to improve the degree of scoring agreement by moderation among the raters, allowing the reliability of OSCEs in clinical practice to be validated.

## Figures and Tables

**Table1:** Scoring rubric for objective structured clinical examinations

Skill scoring item	Contents	2 points	1 point	0 points
1	The examinee briefly describes the movement of shoulder abduction.	The examinee performs all the tasks.	The examinee provides an explanation to the patient in technical terms.	The examinee performs none of the tasks.
2	The examinee places the patient in the measurement position and relaxes the patient’s paralyzed shoulder joint muscles.	The examinee performs all the tasks.	The examinee places the patient in the measurement position but cannot relax the paralyzed shoulder joint muscles.	The examinee performs none of the tasks.
3	The examinee explains and demonstrates the goniometer used in the test.	The examinee performs all the tasks.	The examinee performs only one of two tasks: provides an explanation of the angular scale or performs a demonstration of joint movement.	The examinee performs none of the tasks.
4	The examinee confirms the pain by performing active and passive abduction of the patient’s non-paralyzed shoulder joint.	The examinee performs all the tasks.	The examinee tests the abduction movement of the patient’s shoulder but does not check for pain.	The examinee performs none of the tasks.
5	The examinee abducts the patient’s shoulder and checks the patient’s posture, scapular motion, and presence of subluxation.	The examinee performs all the tasks.	The examinee checks the patient’s shoulder abduction motion but does not evaluate posture, scapular movement, and subluxation.	The examinee performs none of the tasks.
6	The examinee suppresses the compensatory movement of the patient.	The examinee performs all the tasks.	The examinee has inadequate ability to suppress the compensatory movement of the patient.	The examinee performs none of the tasks.
7	The examinee externally rotates the patient’s shoulder joint at 90 degrees of abduction.	The examinee performs all the tasks.	The examinee externally rotates the patient’s shoulder joint, but not at 90 degrees of abduction.	The examinee performs none of the tasks.
8	The examinee abducts the shoulder joint while guiding the patient’s scapula.	The examinee performs all the tasks.	The examinee abducts the shoulder joint while guiding the patient’s scapula, but the abduction is inadequate.	The examinee performs none of the tasks.
9	The examinee checks the patient’s final position of abduction and prepares a goniometer.	The examinee performs all the tasks.	The examinee checks the patient’s maximum range of motion for shoulder abduction but is unable to prepare a goniometer.	The examinee performs none of the tasks.
10	The examinee adjusts the angle meter to the basic axis and the movable axis.	The examinee performs all the tasks.	The examinee only aligns the meter to either the basic or moving axis.	The examinee performs none of the tasks.
11	The examinee measures the angle at the maximum range of motion for the patient.	The examinee performs all the tasks.	The examinee only takes measurements in the maximum range of motion or reads the scale correctly in increments of 5°.	The examinee performs none of the tasks.
12	The examinee safely handles the patient’s upper extremities.	The examinee performs all the tasks.	The examinee handles the patient’s upper extremities poorly.	The examinee performs none of the tasks.
13	The examinee compares the paralyzed side with the non-paralyzed side.	The examinee performs all the tasks.	The examinee measures the range of motion on the paralyzed side of the patient but does not compare it with the non-paralyzed side.	The examinee performs none of the tasks.
14	The examinee communicates the measurement results and interpretation to the patient.	The examinee performs all the tasks.	The examinee only gives the patient the measurement results.	The examinee performs none of the tasks.

**Table2:** The scoring agreements for videos 1 to 40

Assessment Video Number	Kappa coefficient		
	Mean	Min.–Max.	SD
1–10	0.49	0.23–0.85	0.21
11–20	0.57	0.25–1.0	0.22
21–30	0.66	0.31–0.91	0.21
31–40	0.82	0.5–1.0	0.19

Min., minimum; Max., maximum; SD, standard deviation
